# A Self-Structure-Enhanced Algorithm for Pig Point Cloud Completion

**DOI:** 10.3390/ani16142237

**Published:** 2026-07-19

**Authors:** Zhankang Xu, Xiangyu Qi, Qifeng Li, Yikai Fan, Simon X. Yang, Zhaoyang Wang, Weihong Ma

**Affiliations:** 1 Information Technology Research Center, Beijing Academy of Agriculture and Forestry Sciences, Beijing 100097, China; xuzhankangs@163.com (Z.X.); qxy@cau.edu.cn (X.Q.);; 2 College of Information Engineering, Northwest A&F University, Yangling 712100, China; 3 Shangrao Municipal Bureau of Agriculture and Rural Affairs, Shangrao 334000, China; 4 Advanced Robotics and Intelligent Systems Laboratory, School of Engineering, University of Guelph, Guelph, ON N1G 2W1, Canada

**Keywords:** precision livestock farming, point cloud completion, point cloud reconstruction, point cloud missingness

## Abstract

Three-dimensional phenotypic data of pigs provide important information for evaluating growth, nutritional status and health, and they are fundamental to precision feeding, performance assessments, genetic selection and intelligent livestock management. However, point clouds acquired in real pig-house environments are frequently incomplete because of occlusion, limited camera viewpoints, surface reflection, sensor noise, etc. To address local missing structures and geometric discontinuities in pig point clouds, this paper proposes a self-structure-enhanced completion method. The method follows a global-to-local two-stage framework. In the global stage, a self-view fusion network (SVFNet) integrates an incomplete point cloud and its three orthogonal self-projected depth maps to generate a coarse complete shape. In the local stage, a self-structure dual generator (SDG) progressively refines and upsamples the coarse result through a structure analysis and a similarity alignment. To address the physical limitation of distinguishing single-view occlusion from true missingness, this paper proposes a visibility-incompleteness mask (VIM) as the primary contribution, which explicitly models both geometric missingness and multi-view visibility. Furthermore, a prior-adaptive hybrid generation (PAHG) strategy is introduced as a secondary enhancement to combine learnable global shape priors with input-adaptive geometric queries. A dataset containing 1042 complete–incomplete pig point cloud pairs with six typical missing patterns was constructed for model training and evaluation. The proposed method achieved an F-Score@1% of 0.653, a CD-L1 of 9.766, and a CD-L2 of 0.353 on the test set, demonstrating a competitive aggregate performance compared with state-of-the-art completion methods, with trade-offs across different geometric metrics.

## Simple Summary

Three-dimensional point clouds can describe the body shape of pigs and are useful for precision feeding, growth monitoring, breeding evaluations and intelligent farm management. In real pig houses, however, point clouds often contain missing regions because animals are partly blocked by fences, cameras can observe only limited views, and depth sensors are affected by noise and reflections. This study proposes a self-structure-enhanced point cloud completion method for pigs. The method first estimates a coarse complete pig shape by combining the incomplete point cloud with self-projected depth maps from multiple views, and then refines the missing regions through a dual-generator structure that uses both the shape structure and local similarity. A prior-adaptive hybrid generation strategy and a visibility-incompleteness mask are further introduced to help the network focus on truly missing and unreliable regions. Experiments on a pig point cloud dataset with six typical missing patterns showed that the proposed method improves geometric completeness and continuity, especially in easily occluded parts such as the back, abdomen and legs.

## 1. Introduction

Pig production is an important component of animal husbandry, and its productivity and management level are closely related to the supply of livestock products and industrial security [1]. Three-dimensional phenotypic data can directly reflect the growth, nutritional status and health of individual pigs and are strongly associated with breeding selection, performance evaluations and precision feeding decisions. Body-size traits such as body length, body height, chest girth and abdominal girth are not only external phenotypic indicators, but also useful references for conformation assessments, breeding matching and growth monitoring [2,3,4].

With the rapid development of depth cameras, RGB-D sensors and three-dimensional reconstruction techniques, computer-vision-based phenotyping has gradually shifted from two-dimensional image analyses to three-dimensional point cloud processing. Compared with two-dimensional images, point clouds describe the spatial geometry of the animal body more comprehensively, adapt better to posture changes and provide more direct shape information for measurement [5]. Nevertheless, in commercial pig houses, fences, limited observation angles, and sensor noise inevitably cause local missing regions and incomplete structures. This incompleteness creates a detrimental causal chain in precision livestock farming: occlusion and sensor limitations lead to incomplete point clouds, which directly cause severe body-size measurement errors. Consequently, these geometric inaccuracies result in incorrect husbandry management, inaccurate feeding dosages, and flawed genetic selection decisions. Therefore, robust point cloud completion is not just a visual enhancement, but a critical prerequisite for reliable precision farming [6,7]. In this context, pig point cloud completion is not only a geometric reconstruction task, but also a prerequisite for reliable phenotypic quantification. If missing regions occur in the back, abdomen, rump or limbs, key body-size traits may be underestimated or distorted, which further affects growth monitoring and precision feeding decisions. Therefore, a completion model for pigs should not only recover a visually plausible shape, but also preserve biologically consistent body structures under farm-induced occlusion and sensor noise.

In livestock and poultry applications, several studies have used traditional geometric priors, symmetry and interpolation-based methods to repair incomplete animal point clouds. For example, spline repair with a smoothing factor has been used to fill holes in cattle point clouds caused by obstacles and limb occlusion, while mirror mapping based on Euclidean transformations has been used to complete single-side cattle point clouds and least-squares deformation upsampling has been introduced to refine missing surface regions. These methods improve local continuity, but usually rely on hand-designed assumptions and are difficult to generalize to complex non-rigid animal shapes [8,9,10].

General point cloud completion has progressed rapidly from early encoder–decoder frameworks (e.g., PCN, TopNet) to advanced transformer-based and progressive refinement methods (e.g., PoinTr, SnowflakeNet, AdaPoinTr) [11,12,13,14,15,16]. These methods have greatly improved global shape inference, local detail recovery and point distribution uniformity. However, these methods are predominantly designed for regular object datasets like ShapeNet. Research on real livestock point clouds—which are non-rigid, biological, and frequently affected by severe physical occlusion—remains insufficient. Pig point clouds in real farms contain large missing regions, posture changes and local surface self-similarity, which impose higher requirements on prior representation, geometric consistency and cross-scene generalization [17,18].

To solve the problems of local missing regions, structural breaks and geometric discontinuity in pig point clouds caused by occlusion, limited viewpoints, reflection and sensor noise, this study proposes a self-structure-enhanced pig point cloud completion algorithm. The main contributions are as follows:

First, a pig point cloud dataset with six typical missing patterns was constructed to approximate real acquisition conditions.

Second, a visibility-incompleteness mask (VIM) was proposed as our primary algorithmic contribution to address the physical challenge of distinguishing single-view occlusion from true missingness. VIM guides the network to focus on unreliable regions, thereby significantly improving boundary continuity and detailed geometric recovery.

Third, a prior-adaptive hybrid generation (PAHG) strategy was introduced as a supplementary module to combine global shape priors with local input features, dynamically adapting to different missing structures.

The animal study protocol was approved by the Institutional Animal Care and Use Committee (or Ethics Committee) of the Information Technology Research Center, Beijing Academy of Agriculture and Forestry Sciences (protocol code: AW-2025-05-01 and date of approval: 20 May 2025).

## 2. Materials and Methods

### 2.1. Data Acquisition

The experimental data were collected from July to November 2025 over a five-month period at a large-scale pig farm in Longyao County, Xingtai City, Hebei Province, China. Duroc x Landrace x Yorkshire crossbred pigs were selected as the research objects. The data acquisition environment was a standard pen-type pig house equipped with lighting facilities and automated feeding devices. During the experimental period, the indoor temperature ranged from approximately 15 °C to 30 °C. To maintain consistent data quality, the illumination in the acquisition area was controlled as stably as possible, and strong shadows and reflections were reduced. The floor and fence layout were kept fixed to reduce background interference during subsequent processing.

A total of 440 pigs were sampled, covering a body-weight range of 25–150 kg and including the main growth stages from early growth to middle and late fattening. The dataset included different postures, such as slight head turning and short walking pauses, as well as individual body-shape differences, which increased the data diversity and helped the model learn robust structural representations.

To reduce the influence of feeding behavior on abdominal girth, chest girth and hip girth, and to keep the pigs in a relatively stable natural standing posture, body-size and point cloud data were collected before daily feeding from 6:30 to 8:30 a.m. During this period, the gastrointestinal contents were relatively low and the animal activity was more concentrated, which facilitated the acquisition of stable and clear point clouds.

**Figure 1:**
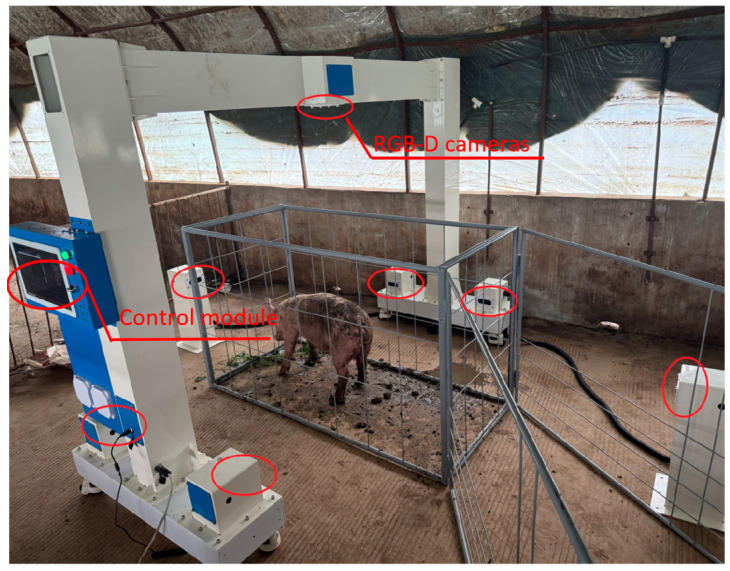
Data acquisition system: seven-view collection in the pig-house passage.

As shown in Figure 1, the seven-view point cloud acquisition device was designed for the automated and standardized collection of pig body morphology data. The system consisted of seven Femto Bolt depth cameras (Orbbec, Shenzhen, China) installed at fixed viewpoints around the pig-house passage to capture the pig body from multiple directions. The cameras were operated in WFOV 2 × 2 BINNED mode, with a field of view of 120° × 120°, an effective measurement range of 0.25–2.88 m and a frame rate of 30 fps. The device was installed in a pig-house passage with a 1.5 m wide fenced corridor to guide pigs through in a single line. The fence bars were approximately 1 cm in diameter and had limited influence on the point cloud completeness.

The original purpose of the device was to support the automated measurement of pig body morphology and body-size traits. To improve the usability of the collected point clouds, data were acquired when the pigs were observed to be in a relatively normal standing posture. During acquisition, the operator monitored the real-time point cloud or depth-camera interface and manually triggered data collection by clicking the acquisition button when the pig body posture was suitable. After triggering, the seven depth cameras were activated through an internal hardware synchronization unit integrated in the acquisition device, enabling multi-view point clouds to be captured within the same acquisition time window. This manual triggering strategy reduced invalid samples caused by walking, turning, bending or severe body occlusion, while the internal synchronization ensured temporal consistency among the seven views.

Each pig passed through the passage multiple times to obtain point clouds under different natural movement states. After acquisition, samples with severe motion, an abnormal posture or obvious incompleteness were excluded. Only XYZ coordinates were recorded, without RGB color information, to reduce the storage and computational cost and to minimize the influence of illumination variation.

**Figure 2:**
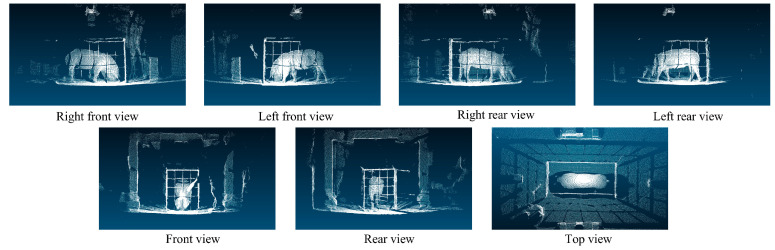
Initial seven-view point clouds obtained from the depth cameras.

**Figure 3:**
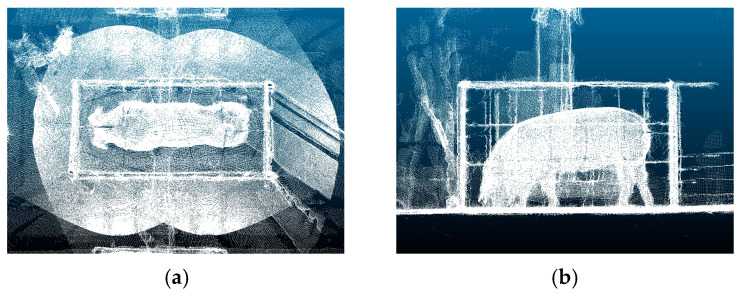
Fused seven-view point clouds. (**a**) Top view; (**b**) side view.

Figure 2 shows the initial point clouds captured from the seven depth cameras before multi-view fusion, including the front, rear, top and oblique views. Compared with three-view data [18], the seven-view point clouds provided higher coverage and spatial continuity in easily occluded regions such as the abdomen, the inner sides of the limbs, the head and the rump. Although structural occlusion from the fences still existed, the narrow fence design and overlapping regions among multiple views enabled partial compensation of the occluded areas, producing nearly complete pig body point clouds after fusion. The fused seven-view point cloud is shown in Figure 3. Compared with sparse-view acquisition, the fused result provides better spatial coverage of the back, abdomen, head, rump and limb regions.

### 2.2. Data Processing

After point cloud segmentation separated the pig body from the environment, relatively complete and clean pig body point clouds were obtained. These seven-view fused point clouds were used as the ground truth, and the corresponding incomplete point clouds were generated as model inputs. Multiple missing patterns were artificially constructed to simulate common incomplete point cloud conditions during real acquisition.

**Figure 4:**
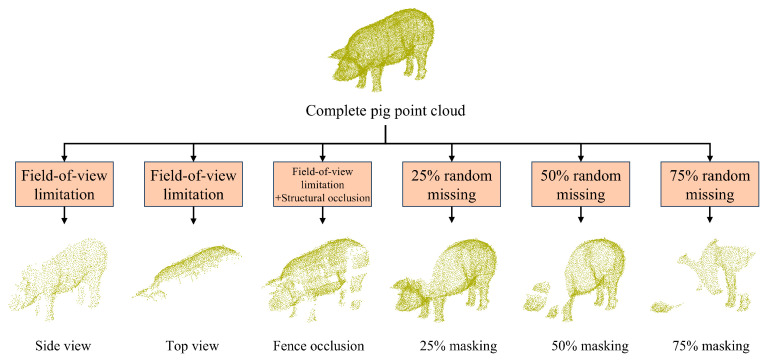
Six typical missing patterns used to construct incomplete pig point clouds.

Figure 4 illustrates the six representative missing patterns used to construct incomplete pig point clouds for supervised training and evaluation. Six typical missing patterns were designed: (1) three-view fence occlusion missingness, where regularly distributed rod-like structures were introduced to simulate fence occlusion; (2) single-side-view missingness, where only visible points from one side view were retained and random point dropping was added; (3) top-view missingness, where visibility screening from an overhead view caused the lower body to be missing; (4) 25% random missingness, generated by deleting continuous spatial regions to form mild missingness; (5) 50% random missingness, corresponding to moderate missingness; and (6) 75% random missingness, representing severe structural missingness.

In implementation, the point clouds were first cropped according to field-of-view constraints and a visibility analysis, similar to view-frustum-based visibility modeling in computer graphics. Then, occluding objects with specific geometric structures were constructed, and the spatial relationships were used to determine the occluded regions. Random noise perturbation and point dropping were also introduced to make the generated data closer to the actual acquisition conditions. For random missingness, continuous spatial regions were removed instead of simple uniform random sampling, which generated structurally coherent missing regions and better-matched missing patterns caused by occlusion or sensor failure.

The final dataset contained 1042 complete–incomplete seven-view point cloud pairs. The training, validation and test sets contained 834, 104 and 104 pairs, respectively. For each complete point cloud, six incomplete inputs were generated according to the six missing patterns, forming paired data for supervised completion. These six missingness patterns were used to construct paired complete–incomplete samples for supervised training, validation and testing, and can also be regarded as a form of structured data augmentation to improve model robustness under diverse incomplete conditions. They did not directly determine the network architecture, and no pattern-specific branches, pattern-specific loss functions or pattern-specific training strategies were introduced. The model was trained jointly on samples generated from all six missingness patterns to learn a unified completion function. The main quantitative evaluation was conducted on the mixed test set containing all six missingness patterns, aiming to assess the overall robustness of the proposed method under heterogeneous missing conditions.

### 2.3. Proposed Method

The objective of pig point cloud completion is to recover a complete and geometrically continuous pig body point cloud from an incomplete input point cloud. In this study, the incomplete point cloud is denoted as P_in, and the corresponding complete point cloud used as the ground truth is denoted as P_gt. During training and evaluation, P_in was sampled to 2048 points, while P_gt was sampled to 16,384 points. Each point represents the three-dimensional coordinates of a surface point on the pig body. The proposed method follows a global-to-local completion strategy. In the global stage, the incomplete point cloud is first projected into three orthogonal self-view depth maps. These depth maps provide complementary two-dimensional structural cues from different virtual viewpoints. Meanwhile, the original incomplete point cloud is processed by a three-dimensional encoder to extract global geometric information. The self-view fusion network (SVFNet) integrates the three-dimensional point-cloud feature and the two-dimensional self-view features to infer a coarse complete pig shape. In the local refinement stage, the self-structure dual generator (SDG) progressively refines the coarse result. The SDG uses two complementary cues: a structure analysis based on missingness-aware global shape information and a similarity alignment based on local geometric patterns observed in the input point cloud. The final output is a dense completed point cloud with improved structural continuity in missing regions such as the back, abdomen, rump and limbs. To improve clarity, the major modules of the proposed framework are summarized as follows. SVFNet is responsible for global coarse completion. PAHG is embedded in SVFNet and is used to generate coarse points by combining learnable shape priors with input-adaptive geometric queries. VIM is used in the SDG refinement stage to estimate the reliability or missingness confidence of each point and guide the network to focus on unreliable regions. The whole network is trained end-to-end using paired complete–incomplete pig point clouds.

#### 2.3.1. Overview of the Proposed Framework

**Figure 5:**
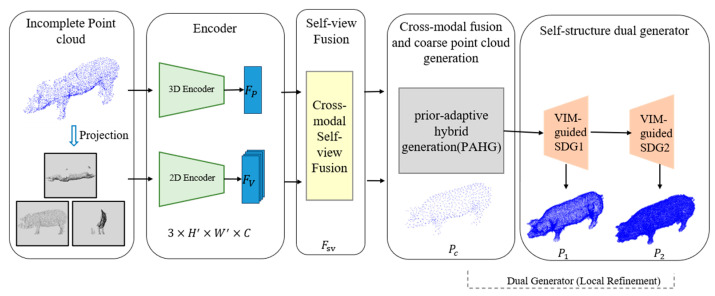
Network architecture of the proposed pig point cloud completion method.

In real pig-house scenarios, point cloud acquisition is affected by occlusion, single-view or sparse-view observation, reflection and range limitation, which often leads to large structural gaps and sparse local noise. These problems reduce the geometric consistency of subsequent three-dimensional reconstruction and body measurements. The proposed method performs effective inference and reconstruction of missing regions to improve the completeness and quality of pig point clouds. The overall network architecture of the proposed pig point cloud completion method is presented in Figure 5.

The network follows a global-to-local two-stage strategy. A cross-modal feature fusion module first generates a global coarse point cloud, and a dual-path refinement module then progressively upsamples and repairs the details. This paradigm is related to PointSea [19], which uses self-projected multi-view depth maps in the global stage and a dual generator based on a structure analysis and a similarity alignment in the local stage.

As shown in Figure 5, the proposed network aims to generate a high-quality complete pig point cloud from a partial low-resolution input point cloud. The network input consists of the partial point cloud, three virtual camera positions corresponding to three orthogonal self-projection views, and the associated self-projected depth maps. The overall architecture contains two main stages: a global self-view fusion stage and a local self-structure refinement stage. In the global stage, the incomplete point cloud is processed by a 3D encoder to extract global geometric features, while the three self-projected depth maps are processed by a 2D encoder to extract view-dependent structural features. SVFNet then performs intra-view and inter-view feature fusion to integrate the 3D global representation and the 2D self-projected depth features. The prior-adaptive hybrid generation module further combines learnable fixed queries with input-adaptive queries to generate a coarse complete point cloud. In the local refinement stage, the coarse point cloud is passed to two cascaded SDG modules, where each SDG contains a structure analysis unit, a similarity alignment unit and a path-selection module. These components jointly predict point-wise coordinate offsets, adaptively fuse dual-path features and progressively refine the missing regions. The final output is a dense and geometrically continuous completed pig point cloud.

**Figure 6:**
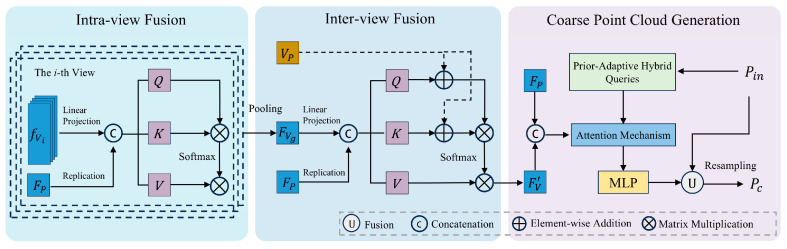
Cross-modal fusion and coarse point cloud generation module.

**Figure 7:**
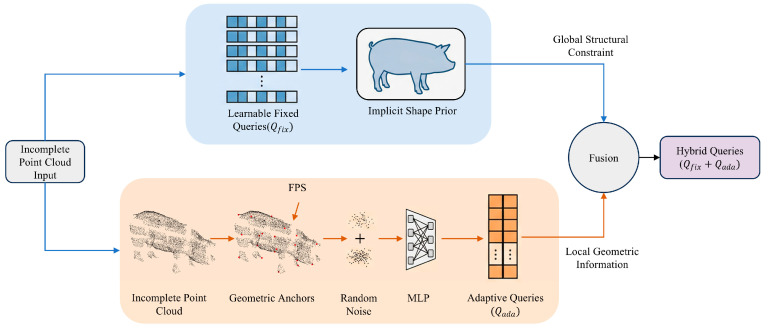
Prior-adaptive hybrid query used in PAHG.

**Figure 8:**
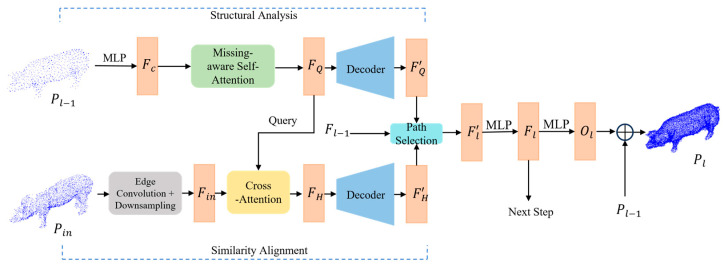
Self-structure dual generator (SDG).

**Figure 9:**
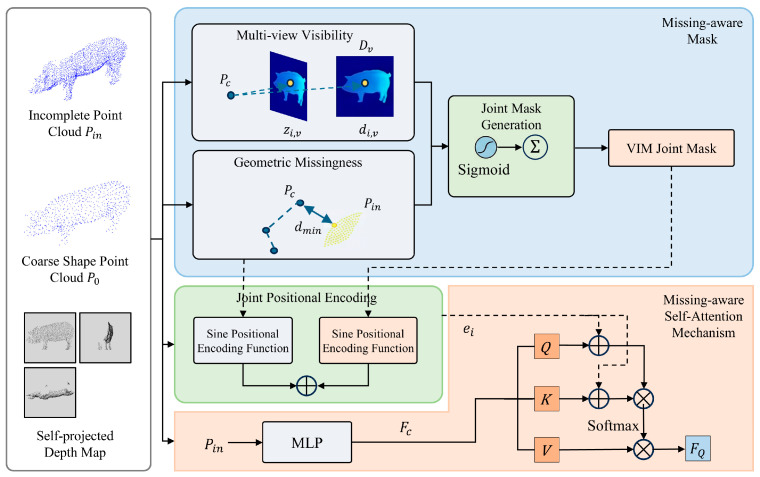
Missingness-aware self-attention mechanism based on VIM.

To clarify the hierarchical relationship among the figures, Figure 5 presents the complete end-to-end architecture, whereas Figure 6, Figure 7, Figure 8 and Figure 9 provide nested expansions of selected modules rather than alternative or repeated network architectures. Specifically, Figure 6 expands the cross-modal self-view fusion and coarse point cloud generation stage shown in Figure 5. Figure 7 further expands only the prior-adaptive hybrid queries block in Figure 6. Figure 8 illustrates the internal architecture of a single SDG block, in which VIM is incorporated into the structure analysis path through the missing-aware self-attention module. This SDG block is applied twice in the cascade in Figure 5. Figure 9 further expands the missing-aware self-attention block in Figure 8 and shows how VIM is generated and injected into the attention computation.

#### 2.3.2. Self-View Fusion Network

SVFNet first extracts a global feature from the point cloud using a point-based 3D backbone and extracts two-dimensional features from the three orthogonal depth maps using a CNN backbone. The direct concatenation of 2D and 3D features is insufficient because of the representation gap between modalities. Therefore, a two-stage feature fusion module was designed to exploit cross-modal information.

It is important to clarify that, while the physical data acquisition employs a seven-camera setup, the SVFNet relies solely on three orthogonal self-projected depth maps generated synthetically from the input point cloud. Self-projected multi-view depth map generation: The depth maps are generated only from the geometry of the input point cloud by projecting it from controlled viewpoints. No additional color images or strict camera intrinsic calibration are required, which is particularly suitable for pig-house environments with variable lighting and frequent occlusion. For each view v, the corresponding rotation matrix $R_{v}\in R^{3\times 3}$ and observation distance $d_{v}$ are defined, and each point $p_{i}$ in the point cloud is transformed into the camera coordinate system:

(1) $$p_{i,v}^{\mathrm{cam}}=R_{v}p_{i}+\left[\begin{array}{c} 0\\ 0\\ d_{v} \end{array}\right],$$

where $R_{v}$ determines the viewing direction, such as top, left or right, and $d_{v}$ controls the distance between the camera and the object to ensure complete projection. Orthographic projection is then used to map the camera coordinates to the image plane:

(2) $$u=\left\lfloor \frac{p_{x}^{\mathrm{cam}}}{\alpha }+\frac{W}{2}\right\rfloor ,$$

(3) $$w=\left\lfloor \frac{p_{y}^{\mathrm{cam}}}{\alpha }+\frac{H}{2}\right\rfloor ,$$

where (u,w) are pixel coordinates, W and H are the width and height of the depth map, and α is a scale factor. If α is not specified, it is automatically determined so that the projected points occupy approximately 80% of the canvas, avoiding both edge clipping and excessive compression. A Z-buffer strategy assigns each pixel the depth value of the nearest point, and interpolation or filling is used to obtain continuous and smooth depth maps.

Dual-branch encoder: The encoder includes a 3D point cloud branch and a 2D depth map branch. Considering the uneven density and noise in real pig-house point clouds, PointMLP was adopted as the 3D feature backbone. Compared with PointNet++, PointMLP uses residual MLP modules to model local geometry with a simple architecture, a strong representation ability and a high computational efficiency. The 2D branch follows the PointSea design and uses ImageNet-pretrained ResNet-18 to extract features from 224 × 224 projected depth maps. Color mapping is not required, and the depth maps provide stable local structural representations for 2D-3D fusion.

In SVFNet, the incomplete point cloud and its self-projected depth maps are encoded through two parallel branches. The 3D branch uses PointMLP to extract a global geometric representation from the incomplete pig point cloud. This feature mainly describes the overall body morphology, including the relative spatial distribution of the back, abdomen, head, rump and limbs. The 2D branch uses ResNet-18 to extract view-dependent structural features from the three orthogonal self-projected depth maps. These features provide complementary shape cues from different virtual viewpoints. The extracted 3D and 2D features are then integrated through intra-view and inter-view fusion. In intra-view fusion, the global 3D feature is injected into each view-specific 2D feature, allowing each projected view to perceive the overall pig body structure rather than only local depth-map patches. In inter-view fusion, information is exchanged among the three orthogonal views so that complementary structures observed from different virtual viewpoints can be jointly modeled. The fused feature is subsequently used for coarse point generation.

Figure 6 provides a detailed expansion of the cross-modal self-view fusion and coarse point cloud generation stage in Figure 5. The notation F_P_ is used consistently to denote the global geometric feature extracted by the 3D encoder. The notation F_V_ in Figure 5 represents the collection of the three view-specific features, extracted from the self-projected depth maps.

For each view i, intra-view fusion injects the replicated global point-cloud feature F_P_ into the corresponding view feature f_vi_. The resulting view-enhanced features are subsequently pooled and exchanged in the inter-view fusion stage. The viewpoint embedding V_p_ is introduced into the query and key branches to preserve the viewpoint identity and model the spatial relationships among the three orthogonal views.

During coarse point cloud generation, F_P_ and F_V′_ are concatenated to form the cross-modal context feature. The hybrid query Q_0_, generated by PAHG as detailed in Figure 7, retrieves relevant geometric information from this fused feature through the attention mechanism. The attended features are mapped to three-dimensional coordinates by an MLP. The generated points are then merged with the original incomplete point cloud P_in_ and resampled to obtain the coarse complete point cloud P_c_. Therefore, P_c_ is the output of Figure 6 and serves as the input P_0_ of the first VIM-guided SDG shown in Figure 5.

The existing coarse point generation methods usually initialize predictions with fixed queries or seed points. Although these methods provide a basic shape outline, they are weak in adaptive adjustment to the local geometry of the input point cloud. To overcome this limitation, a prior-adaptive hybrid generation (PAHG) strategy was proposed. Figure 7 shows the prior-adaptive hybrid query strategy used in PAHG, where fixed learnable queries and input-adaptive queries are fused for coarse point generation. The core of PAHG is a hybrid query that combines learnable fixed query vectors and input-adaptive query vectors. The fixed queries encode an implicit statistical shape prior of pig point clouds and maintain global structural completeness under severe missingness. The adaptive queries are generated by selecting representative points from the input point cloud through farthest point sampling, concatenating them with Gaussian noise and mapping them to the feature space using an MLP:

(4) $$Q_{ada}=MLP([P_{s};\;\epsilon ]),\epsilon ∼N(0,\sigma_{q}^{2}),$$

The noise term weakens strict dependence on the observed point distribution, provides extrapolation capability toward potential missing regions and improves the robustness. The fixed and adaptive queries are fused to obtain the initial query representation:

(5) $$Q_{0}=Q_{fix}+Q_{ada},$$

Figure 7 expands only the prior-adaptive hybrid queries block shown in Figure 6 and does not independently generate the coarse point cloud. The learnable fixed queries Q_fix_ encode an implicit global shape prior, whereas the input-adaptive queries Q_ada_ provide local geometric information conditioned on P_in_. Their fusion produces the hybrid query Q_0_, which is returned to the attention mechanism in Figure 6. There, Q_0_ retrieves geometric information from the fused cross-modal feature, and the resulting features are mapped to three-dimensional coordinates by an MLP. The generated points are subsequently merged with P_in_ and resampled to produce P_c_.

#### 2.3.3. Self-Structure Dual Generator

Figure 8 presents the detailed architecture of a single SDG block shown in Figure 5. Two SDG blocks with the same architecture are applied sequentially in the local refinement stage. At refinement stage l, the current point cloud P_l−1_, the original incomplete point cloud P_in_, and the feature inherited from the preceding stage are processed by the structure analysis and similarity alignment paths. The path selection module adaptively combines the two complementary features and predicts the point-wise coordinate offset O_l_. The refined point cloud is obtained as:

(6) $$P_{l}=P_{l-1}+O_{l},$$

SDG is iterated twice in the network to progressively recover high-resolution geometric structures.

Visibility-incompleteness mask: PointSea introduces a missingness embedding by computing the minimum Euclidean distance from each point in the coarse point cloud to the partial input point cloud and encoding this distance into the attention mechanism. However, distance alone cannot fully distinguish true missingness from reasonable extrapolation and does not use multi-view visual information. Therefore, this study proposes an explicit visibility-incompleteness mask (VIM), which combines geometric missingness and multi-view visibility through a learnable fusion mechanism to produce point-level missing confidence.

(7) $$s_{i}=\underset{y\in P_{\mathrm{in}}}{min}\;\|x_{i}-y{\|}_{2},$$

For each point $x\in P_{l-1}$ in the current coarse point cloud, the geometric missing degree $s_{i}$ is defined as the minimum distance to the partial input point cloud. Points located in missing regions are usually far from the observed points and thus have larger $s_{i}$ values. To complement this geometric cue, the self-projected depth maps are used to compute soft visibility. A point is projected into each view, and its projected pixel coordinate and predicted depth are compared with the observed depth map value:

(8) $${vis}_{i}=\underset{v}{max}exp\left(-\frac{\left|z_{i,v}-d_{i,v}\right|}{\sigma }\right)\cdot I\left[d_{i,v}\right],$$

where indicator function 8 verifies both the pixel validity and depth validity. If a projected point falls in a missing area with no observed depth, the visibility contribution of that view is zero. Multi-view fusion allows the model to judge a point as reliable if it is confirmed by any valid view, thereby distinguishing true pig-body missingness from single-view occlusion. The final mask is obtained by fusing geometric missingness and multi-view visibility:

(9) $$m_{i}=\sigma \left(w_{s}\cdot s_{i}+w_{v}\cdot (1-{\mathrm{vis}}_{i})+b\right)\in (0,1),$$

A larger $m_{i}$ indicates that point $x_{i}$ is more likely to be in a geometrically missing or visually unreliable region, while a smaller value indicates a reliable point. For true missing areas, a large geometric distance and low visibility jointly increase the mask value. For reasonable interpolated points on smooth surfaces, multi-view consistency can keep the mask value low and avoid false judgment.

Relationship between PAHG, VIM and SDG: PAHG and VIM were used at different stages of the proposed global-to-local framework. PAHG belongs to the global SVFNet stage and is responsible for generating the coarse complete point cloud. VIM belongs to the local SDG refinement stage and is used to estimate which points or regions are geometrically missing or visually unreliable.

After PAHG generates the coarse point cloud, VIM was computed by comparing the current coarse or refined point cloud with the original incomplete input point cloud and the self-projected depth maps. It combines two complementary cues: geometric missingness, which reflects the distance between generated points and observed input points, and multi-view visibility, which reflects whether a generated point is supported by valid observations in the self-projected depth maps. The resulting mask was introduced into the structure analysis unit of SDG as a missingness-aware guidance signal.

Therefore, PAHG and VIM are sequentially connected rather than independent prediction branches. PAHG first provides a coarse global structure, and VIM then helps SDG focus the refinement process on unreliable or truly missing regions. Both modules are optimized jointly through the final point cloud completion loss.

Figure 9 provides a detailed expansion of the missing-aware self-attention block located in the upper structure analysis path of Figure 8. VIM is not equivalent to the complete missing-aware self-attention block. Instead, VIM is the mask-generation component shown in the upper blue region of Figure 9. The complete block consists of three sequential operations: VIM generation, joint positional encoding, and VIM-guided self-attention.

Structure analysis unit: Figure 9 expands the missing-aware self-attention block in Figure 8 and clarifies the roles of its three inputs. The current SDG-stage point cloud P_l−1_ is used in two places. First, it is compared with the original incomplete point cloud P_in_ to calculate the geometric missingness s_i_. Second, its points are projected into the self-projected depth maps D_v_ to estimate multi-view visibility. The original incomplete point cloud P_in_ therefore serves as the geometric reference for the observed regions, whereas the depth maps D_v_ provide view-dependent visibility evidence.

The geometric missingness and multi-view visibility are fused by VIM to generate the point-level joint mask m_i_. The geometric missingness s_i_ and VIM mask m_i_ are then transformed by two sinusoidal positional encoding functions and added to form the joint positional encoding e_i_. In parallel, P_l−1_ is mapped by an MLP to obtain the point feature F_c_, from which the query, key and value features are generated. The positional encoding e_i_ is added to the query and key branches before matrix multiplication and softmax normalization. The attention-weighted value feature forms the output F_Q_, which corresponds to the output of the missing-aware self-attention block in Figure 8 and is subsequently passed to the decoder in the structure analysis path.

Similarity alignment unit: The similarity alignment unit uses potential local self-similar structures in the input point cloud to optimize the geometric details of the coarse point cloud. Inspired by point proxies, three EdgeConv layers first extract downsampled point-level features from the input point cloud. Then, a cross-attention mechanism aligns the coarse point features with the input point features by using the coarse features as queries and the input features as keys and values. This process injects locally similar structures from the observed regions into the coarse point cloud and supports the fine completion of missing regions.

Path selection module: The two paths provide complementary information: the structure analysis unit learns pig shape priors, whereas the similarity alignment unit captures local geometric similarity. Their importance varies across missing regions. For example, when an observed region has a symmetric or highly similar counterpart, a similarity alignment is more useful; when a region is severely missing, a structure analysis can provide stronger global guidance. The path selection module uses a point-wise gate to dynamically combine the two features together with the previous offset feature and the missingness-aware global context, thereby selecting the most important information for each point.

### 2.4. Experimental Settings

The input incomplete point cloud was randomly sampled to 2048 points, and the complete ground-truth point cloud was sampled to 16,384 points. In SVFNet, three orthogonal self-projection views were used to generate 224 × 224 depth maps. The 3D branch used PointMLP as the backbone, and the 2D branch used ResNet-18 pretrained on ImageNet. The SDG refinement module was applied twice to progressively increase the point density and repair details. The network was trained using paired complete–incomplete point clouds from the training set, and the validation set was used for model selection. Mainstream completion methods were reproduced under the same dataset and testing conditions for fair comparison.

The proposed model was trained on the constructed pig point cloud dataset using paired complete–incomplete samples. No PointSea-pretrained or ShapeNet-pretrained weights were used for SVFNet, PAHG, VIM or SDG. The ResNet-18 encoder in the 2D branch was initialized with ImageNet-pretrained weights, while PAHG, VIM, SDG and other newly introduced modules were randomly initialized and optimized jointly with the whole network. No separate pretraining or fine-tuning stages were performed. The coarse output generated by SVFNet and the refined outputs generated by SDG were supervised using the complete ground-truth point clouds.

### 2.5. Evaluation Metrics

Three metrics were used to evaluate the completion quality: the F-Score@1%, the Chamfer distance with the L1 norm (CD-L1) and the Chamfer distance with the L2 norm (CD-L2). The F-Score is a threshold-based comprehensive metric that measures the overlap between the predicted point cloud and the ground truth using the harmonic mean of precision and recall. The threshold was set to 1% of the diagonal length of the pig bounding box:

(10) $$\text{F-Score}(d)=\frac{2\cdot P(d)\cdot R(d)}{P(d)+R(d)},$$

CD-L1 measures the average nearest-neighbor distance between the predicted point set and the ground-truth point set using the L1 norm. A lower CD-L1 indicates better global consistency and is relatively robust to noise and outliers:

(11) $$d_{CD-l_{1}}(P,G)=\frac{1}{\left|P\right|}\sum_{p\in P}\;\underset{g\in G}{min}\;||p-g|{|}_{1}+\frac{1}{\left|G\right|}\sum_{g\in G}\;\underset{p\in P}{min}\;||g-p|{|}_{1},$$

CD-L2 uses the squared Euclidean distance and is more sensitive to large positional errors, making it useful for evaluating local reconstruction deviations in important body regions such as the abdomen, rump and limbs:

(12) $$d_{CD-l_{2}}(P,G)=\frac{1}{\left|P\right|}\sum_{p\in P}\;\underset{g\in G}{min}\;||p-g|{|}_{2}^{2}+\frac{1}{\left|G\right|}\sum_{g\in G}\;\underset{p\in P}{min}\;||g-p|{|}_{2}^{2}.$$

## 3. Results

### 3.1. Comparison with Existing Methods

To verify the performance and effectiveness of the proposed method on pig point cloud completion, several mainstream methods were reproduced and compared on the same dataset and experimental settings. The compared methods included FoldingNet, PCN, TopNet, GRNet, PoinTr, SnowFlakeNet, AdaPoinTr and PointSea. FoldingNet learns a folding mapping from a two-dimensional manifold to three-dimensional point clouds; PCN adopts a coarse-to-fine generation framework; TopNet generates point clouds through a tree-structured decoder; GRNet combines voxel grids and point representations; PoinTr models point patches as sequences with a transformer; SnowFlakeNet performs progressive snowflake-like point expansion; AdaPoinTr introduces adaptive queries; and PointSea uses a structure analysis and similarity alignment to model missing regions.

**Figure 10:**
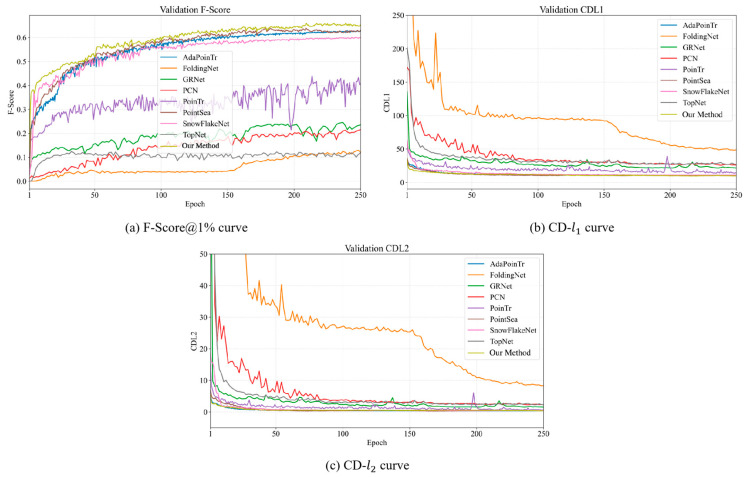
Changes in evaluation metrics during training.

Figure 10 presents the training curves of the evaluation metrics, showing the convergence behavior of the proposed method. The training curves show that the proposed method achieved stable convergence. In the F-Score@1%, the performance increased rapidly and remained stable in later epochs. For CD-L1 and CD-L2, the proposed method showed a faster error reduction and smoother convergence than the comparison methods, indicating better optimization stability and a stronger recovery ability for incomplete pig point clouds.

As shown in Table 1, the proposed method achieved a competitive performance compared with state-of-the-art point cloud completion methods. It obtained the highest F-Score@1% of 0.653 and the lowest CD-L1 value of 9.766. Compared with PointSea, the F-Score@1% increased from 0.630 to 0.653, while CD-L1 decreased from 10.120 to 9.766. However, AdaPoinTr achieved the best CD-L2 value of 0.344, compared with 0.353 for the proposed method. Therefore, the results indicate a small aggregate advantage for the proposed method, together with metric-dependent trade-offs, rather than uniform superiority across all evaluation criteria. Because the per-sample variability and multi-seed variability were not evaluated, these numerical differences should not be interpreted as statistically significant improvements.

**Figure 11:**
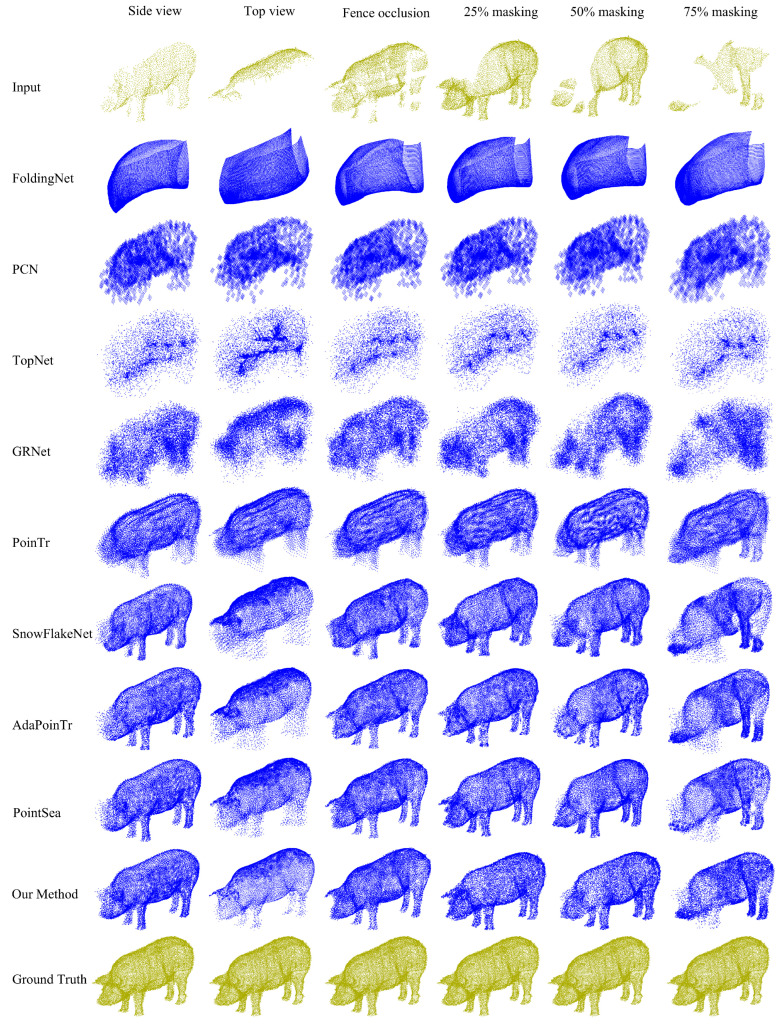
Visual comparison of point cloud completion results from different models.

Figure 11 provides a visual comparison between the proposed method and existing completion models under representative missing patterns. Early mapping-based and hierarchical generation methods, such as FoldingNet and TopNet, performed poorly because they were limited in modeling the global structure and complex missing regions. PCN and GRNet improved completion through coarse-to-fine or voxel-assisted mechanisms, but they still could not fully balance the structural consistency and detailed expression. Transformer-based methods, including PoinTr and its variants, significantly improved the performance, confirming the importance of global context modeling. PointSea further improved the completion through a structure analysis and similarity alignment, but there was still room for improvement in fine modeling and the uncertainty expression of missing regions.

The proposed method improved upon previous work mainly because of PAHG and VIM. PAHG combines a global pig-shape prior with input-specific incomplete point cloud features, allowing the generation process to maintain structural stability while adapting to local geometry. VIM jointly models geometric missingness and multi-view visibility, generates point-level missing confidence and improves the discrimination and reconstruction of missing regions. Visual comparisons show that some comparison methods have structural breaks or blurred details in easily occluded regions such as the back, abdomen and legs, whereas the proposed method generates more complete, continuous and detailed point cloud structures with smoother boundary transitions.

### 3.2. Ablation Study

A systematic ablation study was conducted to evaluate the contribution of key components. Model A used the original PointSea configuration as the baseline, with PointNet++ as the 3D backbone of SVFNet, the original SDG generation strategy and no VIM. Model B replaced the 3D backbone with PointMLP. Model C replaced the original generation strategy with PAHG. Model D replaced the original missingness handling module with VIM. Models E-G combined two improved modules, and the full proposed method combined PointMLP, PAHG and VIM.

As shown in Table 2, the baseline achieved an F-Score@1% of 0.630, a CD-L1 of 10.120 and a CD-L2 of 0.376, indicating a certain completion ability, but insufficient recovery of complex missing structures and details. Replacing the 3D backbone with PointMLP improved all metrics, showing that stronger feature extraction enhances global structural representation. Introducing PAHG also improved all metrics and optimized the distribution quality of generated points. Replacing the missingness module with VIM produced a larger increase in the F-Score and CD-L1, indicating that the explicit modeling of missing regions is especially important for completion.

When modules were combined, the performance improved further. The combination of PAHG and VIM reached an F-Score@1% of 0.647 and a CD-L1 of 9.892, showing that missingness awareness improves local reconstruction while maintaining structural rationality. The combination of PointMLP and VIM achieved an F-Score@1% of 0.651 and a CD-L1 of 9.793, demonstrating synergy between feature extraction and missingness modeling. The full model achieved the best performance, with an F-Score@1% of 0.653, a CD-L1 of 9.766 and a CD-L2 of 0.353. These results show that PAHG improves the structural rationality in generation, VIM enhances the perception of missing regions and PointMLP provides a stronger feature representation basis. Their combination yields the best performance in complex pig point cloud completion.

## 4. Discussion

The proposed self-structure-enhanced pig point cloud completion algorithm achieved high-quality completion under complex occlusion and multi-view missingness by combining a global-to-local framework with PAHG and VIM. The experimental results demonstrated an excellent performance on the test set, but several scientific and engineering issues deserve further discussion.

First, regarding dataset construction, this study built a pig point cloud dataset with six typical missing patterns, including three-view occlusion, single-view missingness, top-view missingness and 25%, 50% and 75% random missingness. This design simulated diverse incomplete point cloud conditions that may occur in practical pig-house environments and provided structured scenarios for model training. However, the current dataset originated from a single commercial farm and a specific crossbred population (Duroc × Landrace × Yorkshire), and therefore, the transferability of the model should be further examined. First, variations in the farm layout, such as the pen width, passage structure, fence density and background materials, may alter the spatial distribution of background noise and affect the initial point cloud segmentation. Second, because the data were collected indoors under relatively stable pig-house conditions without direct sunlight exposure, ordinary illumination variation was expected to have a limited influence on the ToF-based depth acquisition. Nevertheless, strong ambient infrared light, direct sunlight, highly reflective surfaces and multipath interference may still degrade the ToF depth quality in other deployment environments and should be considered in future validation. Third, deviations in the camera placement, including the height, angle and distance to the animal, may change the self-projection geometry and require the recalibration of the depth-map generation parameters. Finally, differences among breeds, genetic lines and growth stages may introduce a geometric domain gap in the body length, back curvature, abdominal contour and limb conformation. Since the PAHG module learns an implicit global shape prior from the training set, applying this prior to morphologically different pig populations may reduce the completion accuracy or introduce biased geometric reconstruction. Future work should validate and fine-tune the framework across diverse farm environments, camera configurations and pig populations.

Second, the method was designed primarily for pig point cloud completion, and the experiments focused on global structural completeness and local geometric consistency. In practical intelligent breeding applications, point cloud completion is closely connected with body-size measurements, behavior analyses and health assessments. Future research can explore the joint training of completion models with downstream phenotyping tasks. A multi-task learning framework may improve both the completion quality and the practical value of reconstructed point clouds for livestock management.

Third, the evaluation metrics used in this study, namely the F-Score@1%, CD-L1 and CD-L2, comprehensively reflect the global structural consistency and local geometric detail. Nevertheless, they may still be limited for evaluating the recovery of specific critical body parts such as the abdomen, back and limbs. Future studies should introduce task-driven metrics, such as the body-size measurement error and key-part geometric error, to quantify the value of the completion results for downstream farm-management tasks more directly.

Fourth, a critical limitation of this study is the inherent bias in the ground-truth data. As noted in Section 2.1, despite fusing seven views, structural occlusion from the narrow fence bars was not entirely eliminated. Consequently, our model was trained to reconstruct a target that is itself physically incomplete. This introduces a systematic bias: the network may learn to replicate these subtle missing regions rather than predicting the true biological surface of the pig, thereby imposing an artificial performance ceiling during evaluation. Therefore, our completion claims must be tempered; the proposed algorithm yields an optimal geometric approximation under constrained acquisition conditions, rather than a perfect anatomical reconstruction.

Finally, the proposed PAHG and VIM modules improve the structure rationality and local detail recovery, but the interpretability of deep point cloud completion remains a challenge. Visualizing missingness confidence, attention maps and the influence of global priors could help explain why the model recovers certain structures and provide a more reliable basis for deployment in precision livestock farming. The model efficiency should also be further considered so that the algorithm can run in real time or near real time on farm-side devices.

## 5. Conclusions

This study proposed a self-structure-enhanced pig point cloud completion algorithm to address occlusion and multi-view missingness in complex pig-house environments. The algorithm adopts a global-to-local two-stage strategy. In the local refinement stage, SDG collaboratively models a structure analysis and similarity alignment to improve the local geometric representation. Crucially, a VIM is proposed to combine geometric missingness and multi-view visibility, successfully overcoming the limitation of distinguishing true missingness from occlusion. Additionally, a prior-adaptive hybrid generation (PAHG) module enhances the stability of coarse generation by combining learnable global priors with input-adaptive queries.

Experiments on the constructed pig point cloud dataset showed that the proposed method achieved an F-Score@1% of 0.653, a CD-L1 of 9.766 and a CD-L2 of 0.353 on the test set. It achieved the best results for the F-Score@1% and CD-L1, while exhibiting a minor trade-off in CD-L2 relative to AdaPoinTr. Therefore, the results support a competitive aggregate performance rather than uniform or statistically significant superiority across all metrics. The method was especially effective in easily occluded regions such as the back, abdomen and legs, generating more continuous and geometrically consistent point clouds. The proposed method provides technical support for high-quality animal point cloud reconstruction and has potential value for intelligent breeding, precision phenotyping and automated body measurement.

## Figures and Tables

**Table 1:** Completion results of different models on the pig point cloud test set.

Model	F-Score@1%	CD-L1	CD-L2
FoldingNet	0.121	48.666	8.349
PCN	0.218	24.935	2.106
TopNet	0.119	26.844	2.251
GRNet	0.235	21.499	1.520
PoinTr	0.432	13.735	0.643
SnowFlakeNet	0.599	11.011	0.418
AdaPoinTr	0.626	10.292	0.344
PointSea	0.630	10.120	0.376
Proposed method	0.653	9.766	0.353

**Table 2:** Ablation study of the contributions of PointMLP, PAHG, and VIM to pig point cloud completion performance.

Model	Baseline	PointMLP	PAHG	VIM	F-Score@1%	CD-L1	CD-L2
A	√				0.630	10.120	0.376
B	√	√			0.636	10.006	0.362
C	√		√		0.634	10.002	0.358
D	√			√	0.644	9.931	0.360
E	√	√	√		0.638	10.007	0.357
F	√		√	√	0.647	9.892	0.358
G	√	√		√	0.651	9.793	0.354
Proposed method	√	√	√	√	0.653	9.766	0.353

## Data Availability

The data supporting the findings of this study are available from the corresponding author upon reasonable request, subject to farm data privacy and research-use restrictions.

## Abbreviations

The following abbreviations are used in this manuscript:

Abbreviation	Definition
SVFNet	Self-View Fusion Network
SDG	Self-Structure Dual Generator
PAHG	Prior-Adaptive Hybrid Generation
VIM	Visibility-Incompleteness Mask
FPS	Farthest Point Sampling
CD	Chamfer Distance
RFID	Radio Frequency Identification
